# Epidemiology of bovine brucellosis in Costa Rica: Lessons learned from failures in the control of the disease

**DOI:** 10.1371/journal.pone.0182380

**Published:** 2017-08-10

**Authors:** Gabriela Hernández-Mora, Nazareth Ruiz-Villalobos, Roberto Bonilla-Montoya, Juan-José Romero-Zúniga, Julio Jiménez-Arias, Rocío González-Barrientos, Elías Barquero-Calvo, Carlos Chacón-Díaz, Norman Rojas, Esteban Chaves-Olarte, Caterina Guzmán-Verri, Edgardo Moreno

**Affiliations:** 1 Servicio Nacional de Salud Animal (SENASA), Ministerio de Agricultura y Ganadería, Heredia, Costa Rica; 2 Programa de Investigación en Enfermedades Tropicales (PIET), Escuela de Medicina Veterinaria, Universidad Nacional, Heredia, Costa Rica; 3 Programa de Investigación en Medicina Poblacional, Escuela de Medicina Veterinaria, Universidad Nacional, Heredia, Costa Rica; 4 Centro de Investigación en Enfermedades Tropicales (CIET), Facultad de Microbiología, Universidad de Costa Rica, San José, Costa Rica; 5 Instituto Clodomiro Picado (ICP), Universidad de CR, San José, Costa Rica; Institut National de la Recherche Agronomique, FRANCE

## Abstract

Brucellosis, caused by *Brucella abortus* is a major disease of cattle and a zoonosis. In order to estimate the bovine brucellosis prevalence in Costa Rica (CR), a total 765 herds (13078 bovines) from six regions of CR were randomly sampled during 2012–2013. A non-random sample of 7907 herds (532199 bovines) of the six regions, arriving for diagnoses during 2014–2016 to the Costa Rican Animal Health Service was also studied. The prevalence estimated by Rose Bengal test (RBT) ranged from 10.5%-11.4%; alternatively, the prevalence estimated by testing the RBT positives in iELISA, ranged from 4.1%-6.0%, respectively. However, cattle in CR are not vaccinated with *B*. *abortus* S19 but with RB51 (vaccination coverage close to 11%), and under these conditions the RBT displays 99% specificity and 99% sensitivity. Therefore, the RBT herd depicted in the random analysis stands as a feasible assessment and then, the recommended value in case of planning an eradication program in CR. Studies of three decades reveled that bovine brucellosis prevalence has increased in CR. *B*. *abortus* was identified by biochemical and molecular studies as the etiological agent of bovine brucellosis. Multiple locus variable-number tandem repeat analysis-16 revealed four *B*. *abortus* clusters. Cluster one and three are intertwined with isolates from other countries, while clusters two and four have only representatives from CR. Cluster one is widely distributed in all regions of the country and may be the primary *B*. *abortus* source. The other clusters seem to be restricted to specific areas in CR. The implications of our findings, in relation to the control of the disease in CR, are critically discussed.

## Introduction

As any other Latin American country, bovine brucellosis is a significant animal health problem and a relevant zoonosis in Costa Rica (CR). Consequently, the disease is of veterinary and of public health relevance. Bovine brucellosis (then recognized as “Bang´s disease”) was clinically described in the Central Valley and in the volcanic highlands at the end of the XIX century, when different breeds of cattle were imported from United States and Europe. The introduction of zebu breeds to CR, mainly from Brazil, initiated at the start of XX century; thereafter, brucellosis was officially recognized as an endemic disease [1–7]. However, cattle exist in CR since 1560, after the introduction of European breeds by the Spanish conquerors from neighboring Nicaragua and Honduras countries. After this, recurrent abortions and reproductive problems of cattle due to brucellosis have been reported until the present time [6].

Although in 1900 the bovine population in CR was close to 350000 [6], brucellosis became just a notifiable disease in 1915, after the first *Brucella* sp. isolation from the blood of a human patient [7,8]. Intervention measures by the Costa Rican National Animal Health Service (CR-NAHS) aimed to the control of the disease in cattle started in 1950 [9]. At that time, reports of “epidemic” abortions, smooth *B*. *abortus* S19 vaccination and agglutination diagnostic tests were the only strategies followed. In 1958, the serological diagnosis of brucellosis in bovine herds was declared obligatory and a national campaign for the control and eradicated of the disease started under voluntary basis with *B*. *abortus* S19 calf vaccination and elimination of the positive reactor animals [10]. At that time the importation of S19 vaccine was under the supervision of the CR-NAHS.

From 1963 to 1965, CR suffered constant ash eruptions of the Irazú volcano, affecting areas of the Central Valley and the surrounding highlands. This natural disaster forced the authorities to abandon the brucellosis program and to allocate the economic resources and personnel in solving the emergency. This natural disaster favored the unrestricted traffic of animals from the affected areas to other regions. Nowadays, and despite the current legislation for traceability of bovine movements nationwide [11], the brucellosis status of the animals is seldom requested and, therefore, infected animals may still be mobilized from one region to another. However, this undisciplined movement of bovines was diminished during the recent ash eruptions of the Turrialba volcano in 2015–2016, when nearly 300 (90%) of the surrounding volcanic herds were tested for brucellosis and the positive animals slaughter before their transfer to safer areas [12,13].

In spite of the efforts, the first attempts for controlling brucellosis failed and in the seventies bovine brucellosis was already widespread in CR [9,10,14]. With a loan from the Inter-American Development Bank, additional actions to implement a brucellosis control program on an obligatory basis were undertaken [14]. Still, those were difficult times for Central America. Although CR did not have internal military conflicts, the critical growing political upheaval against authoritarian regimes in several neighboring Central American nations negatively impacted the country. In addition, during the early eighties CR suffered a severe economic recession. As consequence, the field activities devoted to the control of brucellosis, such as S19 vaccination, test and slaughter considerably diminished [10,15].

Although not implemented, the obligatory basis of the control program remained until 1999, period at which the legislation for the National Bovine Brucellosis Program was finally modified to a voluntary basis by the CR-NAHS in coordination with the livestock producers, the milk industry, other private enterprises and non-governmental organizations [16].

Following, the eradication of brucellosis in United States and Canada with *B*. *abortus* S19 and the corresponding banning of vaccination policies in these countries, rough *B*. *abortus* RB51 vaccine was implemented in CR in 1999 [17]. Although S19 vaccination is still allowed [18], the importation of this smooth vaccine strain was interrupted in 2000 by the CR-NAHS. For all practical purposes the vaccination with S19 was abandoned in the country and in the Central American region [17]. Since 1999, private enterprises, mainly the dairy companies, are devoted to immunize a low number of herds with RB51 vaccine [10,19].

Currently, vaccination and most of the serological testing of the bovines is on voluntary basis. However, CR-NAHS may request testing of the animals for epidemiological surveillance or upon suspicion of brucellosis. By law, all animals depicted as positive must be marked and thereafter slaughter with no further indemnity [19].

**Fig 1:**
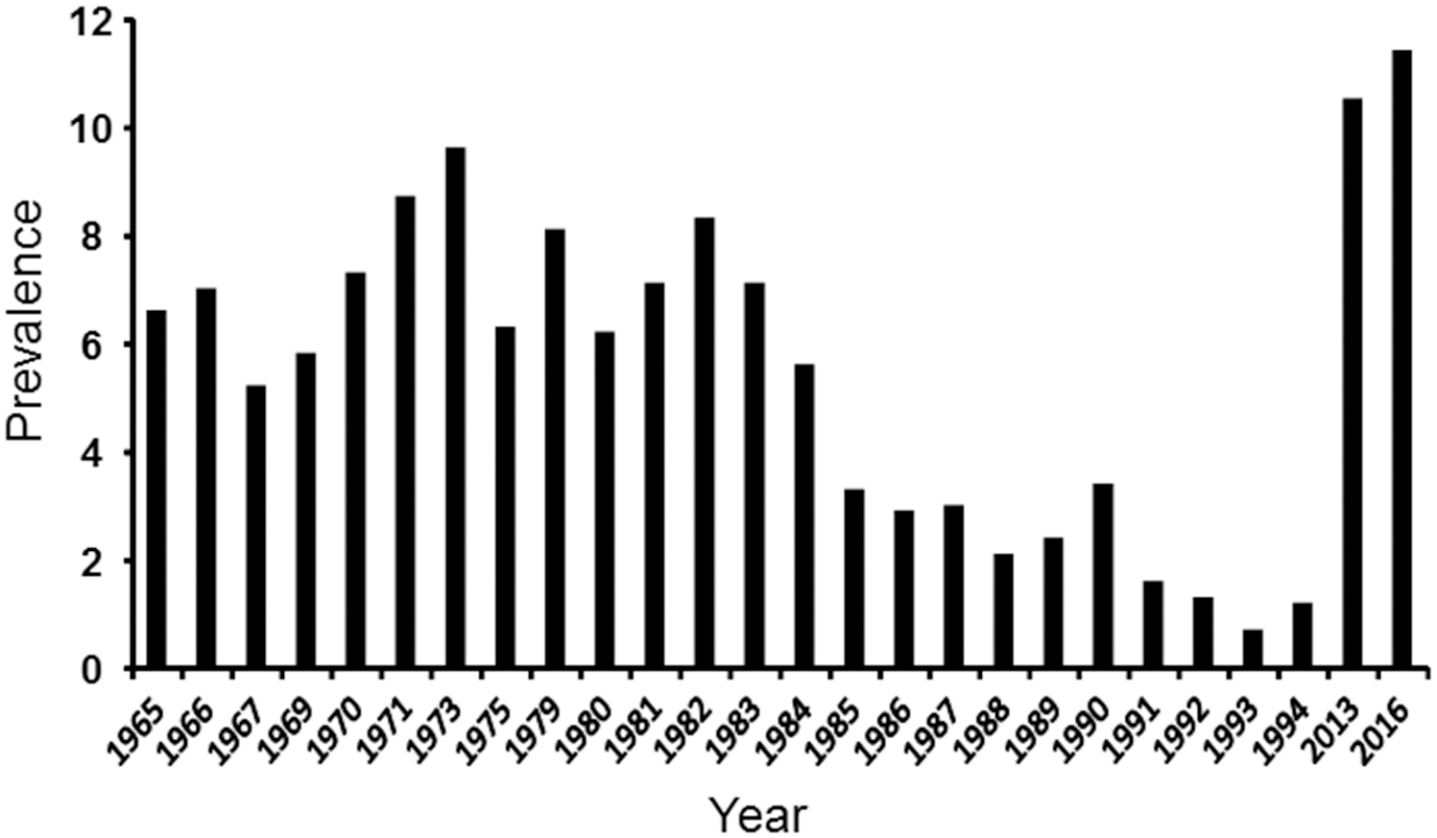
Prevalence of bovine brucellosis in CR during five decades estimated by agglutination tests. The prevalence from 1965–1969 was assessed by tube agglutination; the prevalence from 1970–1986 was assessed by card test in combination with 2- mercaptoethanol agglutination assay; the prevalence from 1987–1994 were estimated by RBT [10,14]. Prevalence values from 2012–2016 assessed by RBT are from this work.

Several studies for estimating the prevalence of bovine brucellosis in CR have been carried out (Fig 1). The last trial before this work was made in 1982 [14]. Therefore, after more than three decades we undertook a new investigation covering all different regions of the country. In this work we describe the distribution of bovine brucellosis, the updated prevalence of the infection and the *B*. *abortus* strains circulating in CR during the lapse of 2012–2016. We also critically discuss the epidemiological implication of our findings in relation to the control programs and the vaccination strategies carried out in CR during the last decades. Distribution and prevalence of brucellosis in other susceptible hosts in CR such as sheep, goats, water buffaloes, pigs, horses, dolphins and humans are described in an accompanying paper [20].

## Materials and methods

### Geography of Costa Rica

CR is a country located in the middle of the Central American isthmus with a surface area of 51100 Km^2^ with Pacific Ocean and Caribbean Sea coastlines of 1016 km and 212 km, respectively. To the North, CR borders with Nicaragua and to the Southwest with Panama. It has been estimated that CR has sixty volcanos, most of them extinct or dormant, but six of them are active. All the volcanos are aligned in a volcanic range were large part of the National parks are located. The country is divided in seven provinces, with a human population close to five million, most of them living in the Central Valley, between the volcanic chain and the mountain range. Socioeconomically the country is divided in six regions: Northern, Central, Brunca, Chorotega, Caribbean Huetar and Central Pacific [21]. The total number of bovines in CR is close to 1.55 million, distributed in about 15000 farms and 50000 herds (S1 Table) [22]. Three different management systems are commonly carried out in the country: beef, dairy and double purpose cattle. Most dairy farms of European breeds (*Bos taurus*) are located in the highlands (from 1000–2500 m); while in the low lands (below 1000 m) are most of the zebu (*Bos indicus*) and mixed breeds (e.g. cebu-holstein cross), used for beef or double purpose production, respectively [22].

### Study population and statistics

The seroprevalence of brucellosis in beef, dairy and double purpose animals were estimated in two bovine populations: i) a non-random sample from sera arriving to the CR-NAHS laboratories for regular diagnosis from herds with history of brucellosis, abortion, reproductive problems, commercial transactions, attendance to exhibitions, exportations and importation of cattle and bovines from herds declared “brucellosis free”, and; ii) a random sample systematically taken in the different regions of CR. To assess both herd and animal prevalence by management system in the later population, a random sample of 250 farms per strata, proportionally allocated by region, were sampled. This sample size was calculated using public access WinEpiscope 2.0 software [23], fitting the following parameters: bovine herd prevalence of 10%, confidence level of 95% and accepted error of 4% for 235 farms; however, it was decided to sample a total of 250 farms per region. A farm was declared positive when at least one serum sample resulted positive. For sample size, the Cannon & Roe formula to demonstrate freedom from/absence of infection, the expected prevalence was adjusted to 15% and a confidence level of 95% [24]. The estimated herd prevalence was founded on the average herd prevalence obtained on pilot study performed in dairy herds in the highlands of the Central Valley of CR. This model does not strictly estimate the within-herd prevalence, but assess the presence of disease. In both studies, the diagnostic strategy was first, screening all bovines by RBT, and then testing of the RBT positives (RBT^**+**^) by iELISA.

The univariate prevalence analysis at the global level and according to production system, were calculated by RBT and RBT^+^+iELISA. In addition, bivariate prevalence for production system by region was also estimated. The prevalence confidence intervals were calculated using beta distribution in the Program @risk [25]. Due to the 99% sensitivity and 99% specificity of the RBT in the absence of S19 vaccination [26,27], a perfection assay was assumed in the analyses.

### Serum samples

**Fig 2:**
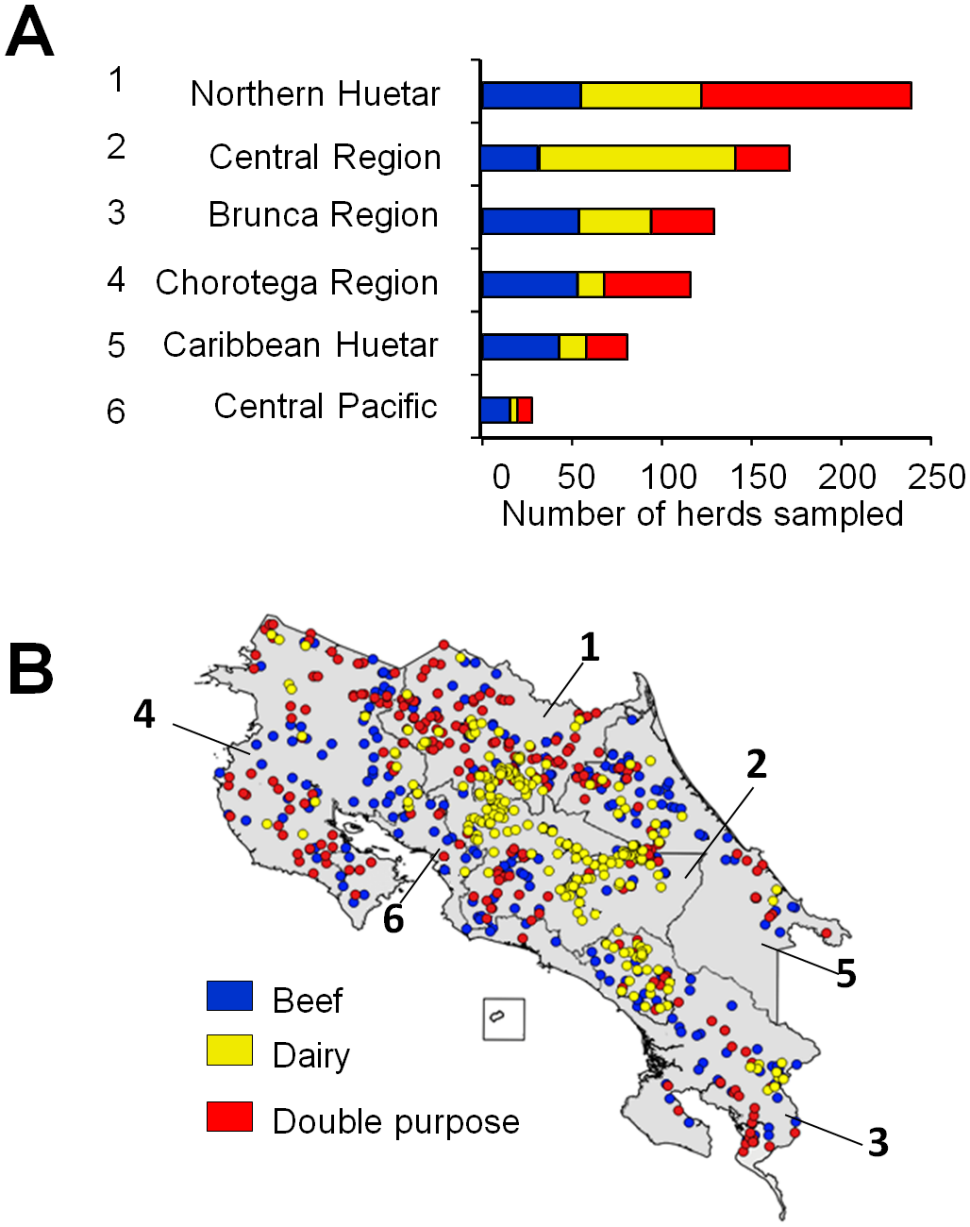
Sampling of cattle farms in the six regions of Costa Rica. (A) A total of 750 farms accounting for close to 18000 cows (2–6 year-old) were sampled during 2012–2013 year period: 250 dairy herds (3902 cows), 254 beef herds (4485 cows) and 261 dual purpose herds (4691cows). (B) Map of CR indicating the different sapling regions (depicted by numbers). Areas of low density of sampling correspond to national parks or protected areas devoid of cattle.

For sampling purposes, the six socioeconomically divided regions of CR were tested (Fig 2). A total of 765 farms accounting for close to 13078 cows (2–6 years of age) were sampled ($\bar{\text{X}}=18$ cows/farm) during 2012-2013-year period: 250 dairy herds (3902 cows), 254 beef herds (4485 cows) and 261 dual purpose herds (4691 cows). In addition, a non-random serum sample of 532199 cows (~35% of the CR bovines) of the six regions ($\bar{\text{X}}=67$ cows/farm) comprising 7907 herds (~16% of CR herds), arriving during 2014–2016 to the laboratories of the CR-NAHS for routine diagnoses, were analyzed. For all purposes, repeated herds were taken into account. For epidemiological purposes, no distinction between breeds or bovine species was considered during the survey.

Blood samples were collected with a syringe or a sterile vacutainer with Z serum clot activator (Vacutainer System, Greiner Bio-one), transported in refrigeration conditions and sera obtained by centrifugation. Each sampled received an individual consecutive number upon arrival to the laboratory. Analyses of the sera were performed within 24–72 hours after collection or arrival at the National Veterinary Laboratories of the CR-NAHS in Heredia, CR, or the Immunology Laboratory of Medicine Veterinary School of the National University, Heredia, CR.

### Information collected for bovine sample

Relevant data concerning the geographical localization, area of the farm and management characteristics of the herd or individual animals, were collected. The information also included the presence of other domestic and wildlife species, veterinary services, reproductive parameters and history of abortion/stillbirth, replacement animals, and history of vaccination against brucellosis. Breed and individual identification was registered.

### Serological tests

Rose Bengal test (RBT) (ID-VET, France) was used as general screening test [28]. Indirect protein A/G ELISA (iELISA) (ID-VET, France) and competitive ELISA (cELISA) (Svanovir, SVANOVA, Sweden) were used as confirmatory tools as described elsewhere [28]. Standardizations of RBT, iELISA and cELISA were performed as described previously [27]. The cut-off values and the specificities and sensitivities of the iELISA and cELISA have been previously established [27,29]. All bovine sera samples were initially screened by RBT and the positives then tested by iELISA and cELISA.

### Culture conditions and *Brucella* identification

The following strains obtained from PIET/CIET strain collections were used as controls for biochemical and molecular studies: *Brucella ceti* Atlantic dolphin type (B14/94), *B*. *ceti* Atlantic porpoise type (B1/94), *Brucella pinnipedialis* seal type (B2/94), *Brucella abortus* 2308W (biovar 1 virulent reference strain), *B*. *abortus* S19 (biovar 1 reference vaccine strain), *Brucella melitensis* Rev1 (biovar 1 reference vaccine strain), *Brucella suis* (S2 biovar 1), *Brucella canis* (CR206-10; CR isolate), *Brucella neotomae* 5K33 (reference strain), *Brucella ovis* PA (virulent reference strain) and *Brucella microti* (CCM4915, reference strain).

According to the National Brucellosis Control Program of the CR-NAHS of the Ministry of Agriculture and Livestock Management, all diagnosed seropositive cattle were selected for obligatory culling [19]. Necropsies or sample collection were carried out at the Pathology Department in the Veterinary School of Universidad Nacional, CR and official slaughterhouses. Animal samples included milk and other secretions such as vaginal swabs and semen, reproductive organs, lymph nodes, spleen, kidney and liver. In some cases, aborted fetuses were also collected and sampled. Cultures were done at the Bacteriology Laboratory of the Veterinary School and at the laboratories of SENASA, using non-selective and selective media including blood agar and Columbia agar, supplemented with 5% of dextrose and sheep blood as well as Modified *Brucella* Selective Supplement (Oxoid^®^ (SR0209) and CITA medium [30]. Cultures were incubated in 10% CO_2_ atmosphere at 37°C for at least two weeks. The selected bacterial colonies were subjected to Gram staining, agglutination with acriflavine and acridine orange dyes and tested for urease and oxidase activity, citrate utilization, nitrate reduction, H_2_S production, growth in the presence of thionin (20 μg/mL) and basic fuchsin (20 μg/mL) and uptake of crystal violet according to described procedures [31].

*Brucella* DNA samples from each isolate and control strains were extracted with DNeasy Blood & Tissue kit from QIAGEN ^®^, and stored at -80°C until used. Identification of *Brucella* species was performed by multiple locus variable-number tandem repeat analysis-16 (MLVA16) following standard procedures [32]. *Brucella* control strains were used for validation. The profiles were entered in the database MLVA-NET for the corresponding analysis [33].

### Ethical considerations

The sampling of bovines is part of the National Brucellosis Control Program of the CR-NAHS of the Ministry of Agriculture and Livestock Management [19] and the Law of Reportable Infectious Diseases of the Ministry of Health of CR [34]. Protocols for the use of bovine serum and tissue samples were revised and approved by the ‘‘Comité Institucional para el Cuido y Uso de los Animales de la Universidad de CR”(CICUA 057–16366), and ‘‘Comité Institucional para el Cuido y Uso de los Animales” of the Universidad Nacional, CR (SIA 0434–14 and SIA 0545–15), and in agreement with the corresponding law ‘‘Ley de Bienestar de los Animales”, CR [35], and according to the “International Convention for the Protection of Animals” endorsed by Costa Rican Veterinary General Law on the CR-NAHS [36].

## Results

In CR the CR-NAHS uses RBT as screening tests and iELISA and cELISA as confirmatory assays [37]. Following this, the results obtained in the analysis of non-random and random samples are presented in Table 1. The RBT herd prevalence levels obtained between the non-random and the random samples were 11.4 and 10.5, respectively. When positive RBT sera was tested by iELISA, the estimated herd prevalence values lowered to 6 and 4.1 respectively Comparable prevalence values observed by RBT^+^+iELISA were obtained when RBT positives were tested by cELISA. The confidence limit 95% of the random sample was 3–6, in rounded numbers. Statistical significance comparisons were made among the different management systems, the random and non-random samples and among the various serological assays used. The only result that showed significant difference in RBT was the double purpose herds in the non-random sampling. When positive RBT samples were tested by iELISA, the results of dairy herds from the non-random sampling were significantly different from the other two management systems. Finally, when comparing both samplings procedures, there were significant differences in the results between beef and double purpose cattle (Table 1).

**Table 1:** Herd and bovine brucellosis reactors according to management system and sampling procedures*.

		Management System	Number	RBT (%)	RBT^+^+IELISA
**Non-random sample from** **2014-2016**	**Herds**	Beef	806	90 (11.2) aα	56 (6.9) cδ
		Dairy	4479	431 (9.6) aβ	186 (4.2) dε
		Double purpose	2622	377 (14.4) bγ○	231 (8.8) cζ
		**Total**	**7907**	**898 (11.4)**	**473 (6.0)**
	**Bovines**	Beef	48129	414 (0.9)	320 (0.7)
		Dairy	346326	481 (0.1)	299 (0.1)
		Double purpose	137744	569 (0.4)	463 (0.3)
		**Total**	**532199**	**1464 (0.3)**	**1082 (0.2)**
**Random sample from 2012–2013**	**Herds**	Beef	254	24 (9.4) aα	8 (3.1) cη
		Dairy	250	22 (8.8) aβ	11 (4.4) cε
		Double purpose	261	34 (13.0) aγ	12 (4.6) cθ
		**Total**	**765**	**80 (10.5)**	**31 (4.1)**
	**Bovines**	Beef	4485	33 (0.7)	9 (0.2)
		Dairy	3902	37 (1.0)	15 (0.4)
		Double purpose	4691	90 (1.9)	50 (1.1)
		**Total**	**13078**	**160 (1.2)**	**74 (0.6)**

* Numbers in parenthesis indicate the seroprevalence. Latin alphabet letters (a-c) represent statistical differences of p ≤ 0.05 values, among productive systems, within the sampling method. Greek alphabet letters (α-θ) represent statistical differences of p ≤ 0.05 values among productive systems, sampling methods and according to type of serological test. Letters “a” and “c” within the same column indicate no significant statistical differences among the various management systems and among the non-random and random sampling. On the contrary, letters “b” and “d” indicate that there are significant statistical differences among the various management systems and among random and non-random sampling. Greek letters “α”, “β” and “γ” indicate that there are not significant statistical differences among the RBT results between the non-random and the random sampling. Alternatively, Greek letters “δ”, “ζ”, “η”, “θ” depict significant statistical differences among the results obtained in RBT^+^+iELISA within the sampling method. On the contrary, the Greek letter “ε” indicates no significant statistical differences among the two sampling methods using RBT^+^+iELISA. In the random sample, the confident limit 95% for beef cattle ranged from 1.6–6.1, for dairy cattle from 2.5–7.7, for double purpose cattle from 2.7–7.9 and for the total population of animals from 2.8–5.7. Bovine population in CR shown in S1 Table.

The higher brucellosis RBT prevalence levels in the non-random (Table 2) and random sampling (Table 3) were obtained with double purpose cattle from the Northern Huetar (17.2% and 17%, respectively) and the Caribbean Huetar (20.2% and 13%, respectively) been the latter one the poorer and less developed of CR. In the case of beef cattle, the regions with the highest number of RBT positive herds in the random and non-random samples were also the Northern Huetar (15.1% and 9%, respectively) and Caribbean Huetar (23.9% and 23%, respectively); while the largest numbers of RTB positive dairy herds were detected in the Central (9.5% and 11.9%, respectively) and Caribbean Huetar (15.8% and 20%, respectively) regions. Due to the small number dairy herds in the Central Pacific region, fewer farms were sampled. In spite of this, positive herds were detected. As expected and regardless of the sample method, when positive RBT samples were tested by iELISA, the prevalence values were lower but commensurate to the RBT in the same regions (Tables 1 and 2). The RB51 animal vaccination coverage for five-year period was estimated in 11%, being more frequent in bovines from dairy farms. Although it was not possible to assess the actual numbers or RB51 revaccinated bovines, we confirmed that it was a common and a recommended practice in CR.

**Table 2:** Herd prevalence in a non-random sample according to region and management system 2014–2016.

Region	Beef			Milk			Double purpose			Total		
	N° Herd	RBT	RBT^+^+ iELISA	N° Herd	RBT	RBT+ iELISA	N° Herd	RBT	RBT^+^+ iELISA	N° Herd	RBT	RBT^+^+ iELISA
**1. Northern Huetar**	73	15.1	8.2	1441	11.5	4.1	1048	17.2	11.0	2562	13.9	7.0
**2. Central Region**	74	5.4	2.7	2037	9.5	4.9	262	8.4	6.1	2373	9.3	4.9
**3. Brunca Region**	510	10.9	7.5	380	5.2	0.2	446	13.2	3.3	1336	10.1	4.0
**4. Chorotega Region**	82	6.1	2.4	431	6.0	3.2	365	7.9	5.7	878	6.8	4.2
**5. Caribbean Huetar**	46	23.9	19.6	114	15.8	11.4	396	20.2	15.4	556	19.6	14.9
**6. Central Pacific**	21	14.2	9.5	76	10.5	0.0	105	6.6	1.9	202	8.9	1.9
**Total**	**806**	**11.1**	**7.3**	**4479**	**9.6**	**4.1**	**2622**	**14.4**	**8.8**	**7907**	**11.3**	**6.0**

**Table 3:** Herd prevalence in a random sample according to region and management system 2012–2013.

Region	Beef			Milk			Double purpose			Total		
	N° Herd	RBT	RBT^+^+ iELISA	N° Herd	RBT	RBT^+^+ iELISA	N° Herd	RBT	RBT^+^+ iELISA	N° Herd	RBT	RBT^+^+ iELISA
**1. Northern Huetar**	55	9.0	3.6	67	4.4	3.0	117	17.0	8.5	239	11.7	5.9
**2. Central Region**	32	12.5	0.0	109	11.9	4.6	30	6.7	0.0	171	11.1	2.9
**3. Brunca Region**	54	1.9	1.9	40	10.0	0.0	35	1.9	0.0	129	4.7	0.8
**4. Chorotega Region**	53	9.4	1.9	15	6.6	0.0	48	12.5	2.1	116	10.3	1.7
**5. Caribbean Huetar**	43	23.2	9.3	15	20.0	20.0	23	13.0	4.3	81	16.0	9.9
**6. Central Pacific**	17	11.7	0.0	4	25.0	25.0	8	0.0	0.0	29	10.3	3.4
**Total**	**254**	**10.6**	**3.1**	**250**	**10.0**	**4.4**	**261**	**12.3**	**4.6**	**765**	**10.5**	**4.1**

**Fig 3:**
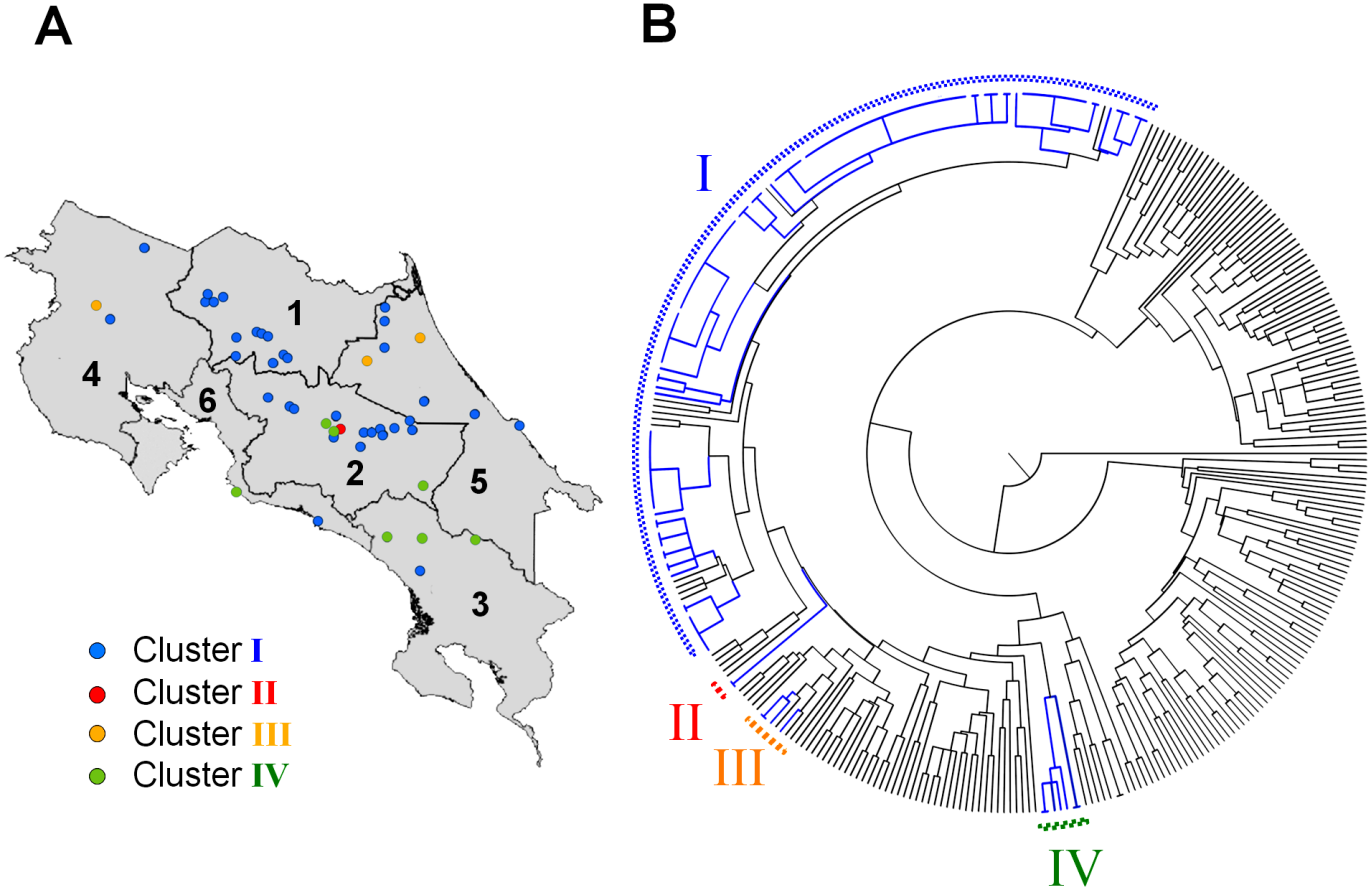
MLVA16 dendogram of *B*. *abortus* isolates from different regions of Costa Rica. (A) Map of CR indicating the different regions from which *B*. *abortus* were isolated (circles). The color of the circles corresponds to the I-IV clusters, respectively. (B) MLVA16 dendogram constructed from the analysis of 107 *B*. *abortus* isolates (depicted in blue lines) are compared with MLVA16 of 219 *B*. *abortus* representative isolates from other latitudes (indicted in black lines). Clusters I to IV are indicted in the figure. S2 MLVA16 genetic profiles for the CR *B*. *abortus* isolates are presented in S2 Table.

*B*. *abortus* has been isolated from dairy, meat and double purpose cattle in all the six regions of CR (Fig 3A). Consistent with previous findings [38,39], *B*. *abortus* biovar 1, 2 and 3 were isolated in different latitudes of CR. *B*. *abortus* MLVA16 clusters were estimated based on differences in three or less tandem repetitions. Following this, the MLVA16 analysis of 326 strains demonstrated that the CR *B*. *abortus* stains (S2 Table) clustered in four main groups (MLVA16 meta-data accessible at http://microbesgenotyping.i2bc.paris-saclay.fr/), suggesting at least four different *B*. *abortus* founders (Fig 3B). Bacteria in cluster one corresponds to the main group, harboring most of the CR isolates; while clusters two, three and four are represented by just a few isolates. Cluster one also includes clinical isolates from aborted fetuses which were identified as *B*. *abortus* RB51 vaccine by Bruce-ladder and supported by MLVA16 (baboCR58 and baboCR57). Clusters one and two are intertwined with *B*. *abortus* from different latitudes. For instance, within cluster one there are isolates from central Europe, USA, India and Brazil. Likewise, cluster three is intertwined with isolates from central Europe, India and Brazil. In contrast, cluster two and four seem to have only representatives from CR. While cluster one is found in all the six regions of CR, cluster three seems confined to the northern areas of the Caribbean Huetar and Chorotega regions and cluster four mainly to the Central and southern areas of the Brunca region. Cluster two is represented just by two isolates confined to the Central region.

## Discussion

We have analyzed the brucellosis herd prevalence in CR by random and non-random methods. The rational of these two schemes is different: while the random sampling is based on a probability theory in which each herd in the population is identified, and has an equal chance of being in the sample; the non-random sampling takes advantage of the samples routinely available for diagnoses. This last non-probability sample is useful for quick and inexpensive studies and for developing hypotheses. When non-random schemes include a large number of individuals and herds within a given population -as it is our case- the values rendered by the analysis may complement the random analysis, and therefore, useful to enforce or deny the hypothesis.

Depending on the strategy employed, brucellosis prevalence varies. For instance, if the RBT results are used as sole parameters, then the prevalence ranges from 10.5% to 11.4%. Alternatively, if the criterion used is the screening of the RBT positives by iELISA, then the prevalence span from 4.1% to 6%. The confidence limit 95% for the random analysis was 3–6%. However, these data deserve careful interpretation. First, detection of RBT false positives due to residual antibodies after vaccination is ruled out in CR. Indeed, the only vaccine used is rough RB51 devoid of O-chain lipopolysaccharide and the vaccine animal coverage in CR is rather low (11%). Under these conditions and in our hands, with a collection of sera from negative and culture positive animals [27], the RBT performs with 99% specificity and 99% sensitivity, values that are commensurate with the findings of other investigators [26]. In spite of this, the RBT may still detect cross reacting antibodies against other bacteria (e.g., *Yersinia enterocolitica* O:9) sharing antigenic determinants with *Brucella*, and then render some false positive reactions [40,41]. Nevertheless, under high brucellosis prevalence, the RBT false positives may have little impact. Moreover, the iELISA and cELISA may also detect cross reacting antibodies [40,41]. Second, the specificity (~98%) and sensitivity (~97%) of the so called “confirmatory assays”, such as iELISA and cELISA [28], depend on the cut off values established [26,27;41]. The current iELISA and cELISA cut off values used in CR and in other Latin American countries were adjusted under S19 vaccination [29], and then intended for detecting antibodies in the infected but not in the S19-vaccinated animals. Finally, the RBT and the iELISA or cELISA may detect different subsets of positive animals [27,41]. This is relevant, taking into account that not all animals were tested by iELISA or cELISA, but just the RBT positives.

Regarding the model used here, there are some drawbacks that deserve attention. Accordingly, a herd was declared positive when at least one serum sample was positive in the RBT following the Cannon & Roe strategy [24]. Sticking to this, it seemed that the average number of 18 animals/herd sampled, became somewhat short. Since the test is not perfect (99% specificity) the probability that 18 bovines in a negative herd, all tested negative, was close to 83%. Then, it follows that the probability that at least one bovine was false-positive ‒and in consequence the whole herd‒, was close to 17%. Likewise, the probability of obtaining a false-positive in given herd decreased with the increased number of positive-diagnosed animals within the group. Testing the RBT positives by iELISA (RBT^+^+iELISA) ensured higher specificity, and the lowest possible prevalence, but not the highest prevalence, which was given by the RBT. It is worth mentioning that while the RBT does not depend on quantitative measures; the iELISA and cELISA depend upon cut-off values, which may vary depending on the epidemiological conditions.

In spite of the limitations of the model and the possibility of cross reactions by the RBT, this test stands as the most reliable assay in the absence of S19 vaccination and low RB51 vaccination coverage [41]. Considering this, it is likely that the RBT herd prevalence depicted in the random analysis is closer to the reality of the country and then, the standing prevalence in case of planning an eradication program in CR. Although the rational of the non-random scheme is different from that of the random sampling, the data in the former somewhat supports the values obtained in the latter.

At least four different *B*. *abortus* MLVA16 clusters are circulating in CR, indicating that the bacterium was introduced more than once in the territory. Cluster one and three are intertwined with isolates from other countries, while clusters two and four have only CR representatives. Since cluster one is widely distributed in all different regions of the country, it seems to be the dominant and the primary source. The relationship of the local strains with *B*. *abortus* from North America, Brazil and Central Europe is not surprising, taking into account that CR cattle came from those lands. The other *B*. *abortus* clusters may be of more recent introduction. It seems to be some association between the MLVA16 clusters and the distribution of the CR isolates. However, in order to unambiguously determine this association and the origin of the clusters, more isolates from different regions are required.

Through the years, efforts have been carried out by the CR animal health authorities to control bovine brucellosis. Unfortunately, these efforts -mainly based in control programs from other latitudes- have been erratic and constantly interrupted [10,14,42,43]. For instance, it is evident that the brucellosis prevalence (estimated by agglutination tests) has increased in relation to that observed in the second half of the eighties and first half of the nineties (Fig 1). During the period of 1978–1985 -after a loan from the Inter-American Development Bank-, a brucellosis control program, known as National Program of Animal Health (PRONASA), was undertaken. PRONASA was intended for ten years and it was coordinated by the CR-NAHS of the Ministry of Agriculture and Livestock Management [10,44,45]. The plan included obligatory *B*. *abortus* S19 vaccination of young replacements, monitoring of abortions, compulsory diagnoses by RBT, 2-mercaptoetanol, rivanol and milk-ring agglutination tests, culling of the serological positive animals with no compensation, and control of animal displacements at specific regional checkpoints [10,14]. During the early years of PRONASA the national vaccine coverage reached close to 43% of bovines and the surveillance was actively taken [14].

Unfortunately, the strong economic recession initiated in 1982 undermined the brucellosis control program. In addition, new political endeavors endorsed the end of PRONASA which was then substituted by PROGASA [44]. In time, this caused the dismantled of the majority of the veterinary field services devoted to the program and, in practical terms, the end of the brucellosis surveillance campaign [10]. By 1984, S19 vaccination reached only one third of the expected coverage [14]. By 1990 the vaccination coverage was less than 15% and finally by the end of the decade, S19 vaccination was interrupted with the subsequent advent of rough *B*. *abortus* RB51 vaccine handled by private hands, mainly by the dairy industry [10,14,19,46]. As stated, the current RB51 vaccination coverage at five year lapse at the animal level is not more that 11%, as estimated in this study and by the annual importation of RB51 vaccine doses to CR [47]. However, this value does not take into consideration revaccination protocols, which are common practices in Costa Rica, and which may interfere with the diagnosis.

Considering the PRONASA 1978–1985 brucellosis control campaign, some errors were made [14,42]. Regardless of the type of vaccine employed, the vaccination coverage in CR has never reached the required levels for adequate herd immunity (at least 70% of coverage). Moreover, the serological testing necessary to detect the brucellosis positive herds reached during the campaigns, was always lower than expected. In addition, the removal of the positive animals was not systematically applied and the economy and political situation of the country did not allow compensation for culling of the reactors. This favored hiding of the positive animals, clandestine sales and transfer of infected cattle to other areas. Moreover, the sole vaccination of young replacements with S19 seemed not enough. Indeed, the logic behind calf S19 vaccination implies extensive survey and constant identification and removal of the positive bovines. However, since testing was not extensively applied, then a significant number of susceptible and infected adult bovines were not identified. All these aspects favored the permanence and spreading of the infection in the country.

One key factor that worsened the problem and deserves attention, concerns to the vaccination policy during the last two decades. In order to “avoid diagnosis confusion" in the detection of *Brucella* infected animals, the regular use of *B*. *abortus* S19 was banned in countries free of bovine brucellosis (e.g. United States and Canada). The Animal Health authorities replaced S19 with RB51 in 2000, before achieving any control of the disease. In addition, the vaccination platform was transferred into private hands mainly to dairy and pharmaceutical companies [18]. We were unable to find documents justifying the rational for these “technical” decisions carried out in CR. This caused the practical obliteration of *B*. *abortus* S19 from the program and the introduction of RB51 as the canonical vaccine [17]. This is not trivial since S19 is the only vaccine that has demonstrated to be successful in eradicating bovine brucellosis [48]. All these events have caused additional problems. Two of them relate to the frequent revaccination, practice known to induce diagnostic problems and increase costs [17, 49–51]. In addition, the unrestricted use of RB51 may promote a “false sense of security”, relaxing the surveillance protocols in the vaccinated herds [52].

Experiences of the various brucellosis eradication programs have demonstrated that the first campaigns were mostly unsuccessful [48]. In countries such as United States, Canada, Australia, New Zeeland or those from Western Europe, eradication of brucellosis was achieved only after the development of joint efforts among the livestock producers, authorities and industry who finally understood the scientific and epidemiological data. They embraced the eradication of brucellosis as their own problem and perceived it as an opportunity to reduce the losses, increase the value of their products and ending with human suffering caused this zoonotic disease [48]. Among the most successful strategies followed by these countries were [48]: i) widespread *B*. *abortus* S19 vaccination coverage of female bovine at risk; ii) single dose immunization of female bovine with complete or reduced S19 vaccine; iii) extensive diagnoses of bovines and herds by sensitive and specific serological assays; iv) obligatory culling of the serological positive animals with compensation actions, and; v) restriction in the traffic of animals from infected areas to free areas.

Although these experiences are relevant, it is unlikely that eradication of bovine brucellosis in CR would be achieved by just applying fixed strategies from other latitudes. Indeed, the eradication of bovine brucellosis is far more complex than just vaccination, testing and slaughtering of the reactors. It is mandatory to consider the idiosyncrasy of each country at the time of initiating campaigns towards the elimination of the disease. For instance, due to the high brucellosis prevalence in CR, immediate slaughtering of all the rectors and confining the herds seem unpractical and not economically feasible. First, it would be necessary to lower the prevalence by limiting the rate of infection and reducing the number of abortions. These may be achieved by extensive and unrestricted vaccination of all female bovines (young and adults) by the conjunctival route with reduced dose S19 vaccine; this, without previous diagnoses and without testing of the animals for two years. Such a strategy—which seems unorthodox−, is known to practically eliminate the clinical disease and to diminish the degree of cattle infection at risk [53]. After few years (e.g. two years), this approach would reduce the prevalence and density of the bacteria in the bovine population to numbers where “a clean” vaccination program of young replacements with S19 (e.g. reduce dose by the conjunctiva route) would be feasible. Then, a serological identification and slaughter of the positive animals might be initiated under more favorable herd infection conditions, allowing some compensation for culling the reactors.

Since the first surveillances performed eighty years ago [7], it is clear that brucellosis remains as a relevant disease of cattle in CR. The steady increase in the brucellosis detection and the consistent isolation of the bacterium in all regions supports the high prevalence and validate the notion that in CR *B*. *abortus* is a source of important economic losses and human health suffering [17,20]. Within this perspective, it seems that the brucellosis conditions prevailing in CR are not unique, and other regions in Latin America display similar vaccination strategies and epidemiological profiles [54–56]. Therefore, our findings are relevant within a broadest context.

Why does after one hundred years of the first isolation of *Brucella* in CR, this small country has not been capable to lower the prevalence and eradicate brucellosis? Certainly, countries about the same size as CR have eradicated brucellosis. Moreover, CR has been able to resolve very complex problems [57–59]. For instance, since 1949 the army was abolished, and since 1970 the natural protected areas of the country cover 26% of the territory of CR. Likewise, the Costa Rican public healthcare system is ranked among the highest in the American Continent. Literacy is also comparatively high for a middle range income country. Regarding the cattle industry, a large part of the milk and meat producers are well organized in cooperatives and associations. Above 97% of the farms are electrified, communicated by roads and the veterinary services attending the farms are well trained [6,36]. It seems, therefore, that in order to achieve brucellosis eradication in CR, joint efforts are necessary among scientists, producers, cattle industry and the government. Without cooperation among these parties, even good intentions and first-class strategies are condemned to failure.

## Conclusions

1. Bovine brucellosis due to *B*. *abortus* is widespread in CR and the prevalence of the disease has increased in relation to the last three decades.
2. In the absence of S19 vaccination, the RBT herd prevalence depicted in the random analysis tends to lay close to the reality and then, the suggested value in case of planning an eradication program in CR.
3. In the absence of S19 vaccination, the iELISA and cELISA used as “confirmatory tests” need to be adjusted to the required levels of sensitivity and specificity to fulfill the brucellosis epidemiological conditions of CR.
4. The vaccination campaigns in CR have never been adequately adopted to increase the herd immunity required to decrease the number of susceptible animals below a desired threshold, for control programs.
5. The vaccination coverage in CR is rather low and revaccination with RB51 is a common practice in CR.
6. At least four different *B*. *abortus* MLVA16 clusters are circulating in CR, indicating that the bacterium has been introduced more than once in the territory. Cluster one -widely distributed in all different regions of the country- seems to be the dominant and the primary *B*. *abortus* source in CR.
7. The brucellosis campaigns have been interrupted due to economic problems, deficient animal health services, absence of personnel and weak political support to technical and scientific concerns.
8. The availability of reliable epidemiological data on bovine brucellosis in all regions of CR establishes a background level to envision strategies for the control of bovine brucellosis in the country.

## Supporting information

S1 Table Estimated number of bovines by geographical region and by management system in Costa Rica (2011–2014). (DOCX)

S2 Table MLVA16 genetic profiles for the CR B. abortus isolates. (XLSX)
