# Supplementary material for: Epidemiology of bovine brucellosis in Costa Rica: Lessons learned from failures in the control of the disease
Source: PLoS One. 2017 Aug 10;12(8):e0182380. doi: 10.1371/journal.pone.0182380 (PMC5552303; doi:10.1371/journal.pone.0182380)
Supplement: S1 Table — (DOCX) [file pone.0182380.s001.docx]

**S1 Table.** Estimated number of bovines by geographical region and by management system in Costa Rica (2011-2014)*.

| **Region** | **Beef** | | **Milk** | | **Double purpose** | | **Total** | |
| --- | --- | --- | --- | --- | --- | --- | --- | --- |
|  | **Bovines** | **Herds** | **Bovines** | **Herds** | **Bovines** | **Herds** | **Bovines** | **Herds** |
| Chorotega | 286451 | 5795 | 19731 | 335 | 77558 | 1498 | 383740 | 9253 |
| Central Pacific | 69743 | 1838 | 2176 | 82 | 10394 | 241 | 82313 | 2621 |
| Brunca | 166588 | 5886 | 11531 | 894 | 37581 | 1099 | 215700 | 9557 |
| Central | 72696 | 3465 | 80244 | 2471 | 19255 | 676 | 172196 | 8021 |
| Northern Huetar | 239908 | 6102 | 82678 | 1407 | 167088 | 3652 | 489673 | 13539 |
| Huetar Caribe | 165596 | 4850 | 5103 | 328 | 21603 | 611 | 192302 | 7023 |
| Total | 1000982 | 27935 | 201463 | 5516 | 333479 | 7776 | 1535924 | 50014 |

*Standard error in the number of bovines of ~9% [22, 60]

22. Ministerio de Agricultura y Ganadería. Sistema integrado de registro de establecimientos Agropecuario-SIREA. Servicio Nacional de Salud Animal-SENASA. 2017. Available from: <http://registrosenasa2.addax.cc/>

60. El Instituto Nacional de Estadística y Censos (INEC). Censo agropecuario 2014. San José, Costa Rica. 2014. Available from: http://inec.cr/agropecuario?title=&shs_term_node_tid_depth=All&field_periodo_tid=All&field_anio_documento_value%5Bvalue%5D%5Bdate%5D=
